# Sickle Cell Trait Causing Splanchnic Venous Thrombosis

**DOI:** 10.1155/2015/743289

**Published:** 2015-06-29

**Authors:** Priyanka Saxena, Pratibha Dhiman, Chhagan Bihari, Archana Rastogi

**Affiliations:** ^1^Department of Hematology, Institute of Liver and Biliary Sciences, Vasant Kunj, New Delhi 110070, India; ^2^Department of Pathology, Institute of Liver and Biliary Sciences, Vasant Kunj, New Delhi 110070, India

## Abstract

Sickle cell trait is considered as a benign condition as these individuals carry only one defective gene and typically have their life span similar to the normal population without any health problems related to sickle cell. Only under extreme conditions, red cells become sickled and can cause clinical complications including hematuria and splenic infarction. Although twofold increased risk of venous thrombosis has been described in African Americans, there is no data available from Indian population. We here report a case of sickle cell trait from India whose index presentation was thrombosis of unusual vascular territory.

## 1. Introduction

Inheritance of only one Hb S allele is termed sickle cell trait (SCT). SCT is considered a generally asymptomatic state with few problems for the individual over a lifetime [1]. On a population basis, it has no discernible impact on life expectancy [2]. Increased red blood cell sickling and polymerization can occur in SCT under conditions of severe tissue hypoxia, acidosis, increased viscosity, dehydration, and hypothermia. In vitro studies have established that heterozygous SCT erythrocytes sickle when the oxygen level is decreased to 2% compared with 4% to 6% for homozygous erythrocytes [3]. Subclinical sickling of red cells probably occurs in most persons with SCT and may explain such complications as splenic infarction at high altitudes, essential hematuria, loss of renal concentrating ability, and sudden death after extreme exertion [4]. Compared with matched controls with normal hemoglobin, these individuals have increases in the measures of coagulation activity [5]. Few studies have shown association of SCT with deep vein thrombosis (DVT) and pulmonary embolism (PE) [6, 7]; there is no study showing an association with splanchnic venous thrombosis. Also these studies are reported from African American community whereas no such analysis has been done in Indian population. We hereby report a case of a patient with sickle cell trait who presented with extrahepatic portal vein obstruction (EHPVO) as his index presentation.

## 2. Case Report

A 24-year-old male presented with chief complaints of abdominal pain for 7 days. There was no history of fever, vomiting, diarrhea, substance abuse, and any prior admissions because of the same complaint. On examination, there is mild pallor, no icterus, and no lymphadenopathy. Abdominal tenderness was present without any organomegaly. On investigations, complete hemogram showed Hb/PCV of 11.0 g/dL/33.2%, RBC count of 5.8 × 10^12^/L, MCV of 76.7 fL, MCH of 24.7 pg, MCHC of 32.2 g/dL, and RDW of 17.7%. Total leucocyte count was 6.1 × 10^9^/L with neutrophil 69%, lymphocyte 20%, monocyte 7%, and eosinophil 4% and platelet count was 271 × 10^9^/L. Peripheral smear findings revealed moderate anisopoikilocytosis with microcytic hypochromic cells. Reticulocyte count was 2.1% and serum LDH was 702 IU/L. Liver and renal function tests were within normal limits. PT/INR was 15.2 sec/1.21. His CECT abdomen revealed occlusive thrombus in main portal vein (MPV) and superior mesenteric vein (SMV) with normal liver architecture. He underwent intravascular thrombolysis with radiologically guided placement of thrombolysis catheter followed by urokinase infusion for 72 hours. Procedure was uneventful without any bleeding complication. Postprocedure CECT abdomen showed patency in MPV whereas no flow could be identified in SMV. He was started on anticoagulation in the form of low molecular weight heparin with warfarin to target an INR of 2-3. In view of splanchnic venous thromboses, thrombophilic work-up was done during follow-up after stopping the anticoagulation for 6 weeks, which showed Protein C of 93.2% (normal range: 70%–140%), antithrombin levels of 75% (normal range: 70%–125%), and Protein S of 65% (normal range: 60%–130%). Factor V Leiden, methylenetetrahydrofolate reductase (MTHFR) C677T, and prothrombin G20210A were negative. In view of microcytic hypochromic picture with erythrocytosis, thinking of polycythemia vera as a differential diagnosis, mutational analysis of JAK2V617F and JAK2 exon12 was done which was negative. Also paroxysmal nocturnal hemoglobinuria can present with EHPVO, and flow cytometry analysis of CD55 and CD59 was done which came out to be negative. As all the prothrombotic markers were negative, we again reassessed the patient and got an HPLC done for microcytosis which showed HbF: 0.8%, Hb S: 35.8%, HbA2: 3.3%, and Hb A: 54.5%. On family screening, mother was found to be with Hb S trait. As in SCT, microcytosis may be associated with coexisting alpha thalassemia [8], and we have done mutational analysis of alpha thalassemia which has not detected any mutation. Iron studies were done which showed serum iron of 60 *μ*g/dL and TIBC of 450 *μ*g/dL confirming the diagnosis of sickle cell trait with iron deficiency. As all the prothrombotic markers relevant to the condition were negative, the cause for EHPVO was attributed to the underlying SCT in this patient. In follow-up, complete recanalization of MPV noted with SMV still shows hypoechoic thrombus on hepatic vein doppler. However, there was no progression of thrombus to other vessels. Presently, patient is asymptomatic and on anticoagulation with INR in therapeutic range.

## 3. Discussion

The importance of reporting this case is to highlight the probable association of SCT with splanchnic thrombosis and that it can be the first presentation of the underlying hemoglobinopathy. Also by reporting this case, the authors want to stimulate clinical studies in Indian population to find out the risk of venous thromboembolism (VTE) especially addressing unusual sites in SCT individuals. Though the risk ratio in SCT has been defined for pulmonary embolism in blacks, there are no such studies from India showing the same correlation.

The prevalence rate of *β* ^S^ gene varies from 0 to 34% in different groups and reaches as high as 40% especially in the tribal groups from India [9]. Traditionally, SCT has been viewed as a benign condition, a nondisease, partially protective against falciparum malaria and without any of the painful episodes characteristics of the homozygous sickle cell disease [10]. However, now there is data derived from case reports or uncontrolled observational studies describing the morbidity of SCT. It ranges from definite complications like renal medullary carcinoma, hematuria, renal papillary necrosis, splenic infarction, exertional rhabdomyolysis, and exercise-related sudden death to probable associations with hyphema, VTE, and fetal loss/demise [11]. Data on risk association between SCT and VTE is predominantly reported in African Americans.

Humphries and Wheby described a case report of a 23-year-old man with multiple episodes of recurrent venous thrombosis in which SCT was the only identified potential risk factor [12]. Austin et al. studied 515 hospitalized black patients and 555 black controls obtained from medical clinics.The prevalence of the S allele was 0.070 and 0.032 for case patients and controls. The major finding of this study was that persons with SCT experienced approximately a 2-fold increased risk of VTE compared with persons with the wild-type genotype, but PE (without DVT) is of a 4-fold increased risk among those with SCT, whereas the risk of DVT (without PE) was not meaningfully increased [6].

Also a case-control study of VTE in African Americans has shown approximately 2-fold risk among subjects with HbAS compared with those with HbAA [7]. Plausible explanation of association of VTE with SCT can be that individuals with sickle cell trait have increases in the measures of coagulation activity including higher levels of d-dimers, thrombin antithrombin complexes, and prothrombin fragment. Monocytes which play an active role in endothelial damage and atherogenesis are also increased in some cases [1]. Also as subclinical sickling occurs in these cases, loss of normal phospholipid asymmetry and the resulting abnormal phosphatidylserine exposure are thought to contribute to the hemostatic perturbations [6].

The main implication of our report is the presentation of SCT with EHPVO in which no other prothrombotic factors were present. As abdominal thromboses can be life threatening, thrombophilic work-up adds to accurate management of the patient. We do not recommend HPLC as a part of prothrombotic work-up but every patient should be assessed accordingly. Although we have managed our case with thrombolysis and anticoagulation, the determination of underlying hemoglobinopathy helped in appropriate counseling of the patient regarding carrier state and in recognition of a SCT family.

To the best of our knowledge, there is no such case reported in the past. Importantly, it should be noted that there is lack of data for the association of VTE with SCT in Indian population which is required so that we can formulate the need of thromboprophylaxis in these individuals.
